# Comprehensive Analysis of Tumor-Infiltrating Immune Cells and Relevant Therapeutic Strategy in Esophageal Cancer

**DOI:** 10.1155/2020/8974793

**Published:** 2020-05-11

**Authors:** Guangrong Lu, Liping Chen, Shengjie Wu, Yuao Feng, Tiesu Lin

**Affiliations:** ^1^Department of Gastroenterology, The Second Affiliated Hospital and Yuying Children's Hospital of Wenzhou Medical University, Wenzhou, Zhejiang 325000, China; ^2^Department of Pharmacy, Sir Run Run Shaw Hospital, School of Medicine, Zhejiang University, Hangzhou, Zhejiang 310000, China; ^3^College of Pharmaceutical Sciences, Wenzhou Medical University, Wenzhou, Zhejiang 325000, China; ^4^Department of Gastroenterology, The First Affiliated Hospital of Wenzhou Medical University, Wenzhou, Zhejiang 325000, China

## Abstract

A growing body of evidence has indicated that behaviors of cancers are defined by not only intrinsic activities of tumor cells but also tumor-infiltrating immune cells (TIICs) in the tumor microenvironment. However, it still lacks a well-structured and comprehensive analysis of TIICs and its therapeutic value in esophageal cancer (EC). The proportions of 22 TIICs were evaluated between 150 normal tissues and 141 tumor tissues of EC by the CIBERSORT algorithm. Besides, correlation analyses between proportions of TIICs and clinicopathological characters, including age, gender, histologic grade, tumor location, histologic type, LRP1B mutation, TP53 mutation, tumor stage, lymph node stage, and TNM stage, were conducted. We constructed a risk score model to improve prognostic capacity with 5 TIICs by least absolute shrinkage and selection operator (lasso) regression analysis. The risk score = −1.86∗plasma + 2.56∗T cell follicular helper − 1.37∗monocytes − 3.64∗activated dendritic cells − 2.24∗resting mast cells (immune cells in the risk model mean the proportions of immune cell infiltration in EC). Patients in the high-risk group had significantly worse overall survival than these in the low-risk group (HR: 2.146, 95% CI: 1.243-3.705, *p* = 0.0061). Finally, we identified Semustine and Sirolimus as two candidate compounds for the treatment of EC based on CMap analysis. In conclusion, the proportions of TIICs may be important to the progression, prognosis, and treatment of EC.

## 1. Introduction

Esophageal cancer (EC) is one of the most common digestive tract cancers; the morbidity and mortality of which is ranked 9th and 6th in all malignant tumors. It is estimated that, in 2018, 508,585 people worldwide would die of EC [1]. Thus, it becomes extremely urgent to find effective treatments. In most cases, for cancers in early stages, the general excision of cancers is chosen as the surgical treatment. However, due to the lack of early symptoms, most affected EC patients lose the optimal opportunity of surgery [2]. Although in recent years, new adjuvant therapy, chemotherapy, and precise radiation therapy for EC have made some progresses, unfortunately, the overall efficacy is still not ideal, and the 5-year survival rate is around 30% [3]. Against this backdrop, identifying other effective treatments is significant for improving the survival rate of EC patients.

Increasing evidence indicates that behaviors of cancers are defined by not only the intrinsic activities of tumor cells but also the tumor-infiltrating immune cells (TIICs) in the tumor microenvironment. TIICs are the heterogeneous immune populations existing in the tumor tissues and which play a key role in host antigen-specific tumor immune response. The cells including T cells, B cells, natural killer (NK) cells, macrophages, dendritic cells (DC), and polymorphonuclear leukocytes all belong to TIICs [4]. In this regard, it is reported in hepatocellular carcinoma (HCC) that the functional interaction of tumor-infiltrating T cells and B cells was contributed to the prognosis of HCC patients through immune activation [5]. In breast cancer, the findings demonstrated that the increased fractions of regulatory T cells and M0 macrophages were linked to a lower pathological complete response rate, shorter disease-free survival (DFS), and worse overall survival (OS) [6]. For EC, the reports similarly have linked the presence of TIICs with its treatment response and outcome. In these stage II+III patients, the densities of NK cells and macrophages found a significant relation with patients' postoperative prognoses [7]. For esophageal squamous cell carcinoma (ESCC), the expression of PD-L1 was positively associated with TIIC density and that was also correlated with worse prognosis [8, 9]. Another evidence indicated that regulatory T cell (Treg) infiltrate presented in the tumor had an association with the pathological response and exhibited a favorable value in predicting cancer-specific survival [10]. Thus, the full analysis of the types and the range of TIICs is a promising strategy to make a huge change for the treatment of EC.

In colorectal cancer, it has more recently been established that the type, density, and location of TIICs within tumor samples are more powerful to predict patient survival than the histopathological methods currently used for its tumor stage [11, 12]. However, for EC, it still lacks a well-structured and comprehensive analysis of TIICs and its therapeutic value. Due to the limitations of methods and techniques, previous studies thus were focused merely on the finite areas of immune response. Recently, with the development of a novel metagene approach of CIBERSORT [13], it is possible to computationally dissect the density of TIICs and then to predict the clinical value in EC. In this study, 20 differential fractions of TIICs were identified in normal tissues and tumor tissues by CIBERSORT. Based on the least absolute shrinkage and selection operator (lasso) regression model, it was revealed that TIICs exhibited potential effect in EC prognosis. Additionally, following the gene ontology (GO) analysis for illuminating the differential enrichment signals between the low- and high-risk groups, we also found candidate compounds for the treatment of EC.

## 2. Materials and Methods

### 2.1. Database

The Cancer Genome Atlas (TCGA, RRID:SCR_003193) expression data of normal and tumor tissues of EC was downloaded from UCSC Xena (https://xena.ucsc.edu/) [14]. Because there were only 6 samples of normal tissues that were eligible, we downloaded the expression data of normal esophagus tissues from Genotype-Tissue Expression (GTEx) data from https://www.gtexportal.org/(RRID:SCR_013042) [15]. After normalization by “Limma” package of R (RRID:SCR_010943) [16], the expression data from GTEx were added into the TCGA, which were unified to log_2_ (FPKM+1) (FPKM, Fragments Per Kilobase of transcript per million fragments mapped) to improve the representation. An indirect validation cohort, GSE19417, containing 76 human esophageal adenocarcinoma tissues and some clinical data, was downloaded from GEO Datasets (https://www.ncbi.nlm.nih.gov/geo/query/) [17].

### 2.2. Assessment of Immune Cell Infiltration

The CIBERSORT (RRID:SCR_016955) algorithm was applied to evaluate the proportions of TIICs in tissues. CIBERSORT was a method that was designed and robustly validated to identify 22 human immune cell phenotypes, outperforming other methods in the matter of noise and unknown mixed content [13]. 22 human immune cell phenotypes were analyzed in the study, including seven T cell types (T cell CD8, naïve T cell CD4, resting T cell CD4 memory, activated T cell CD4 memory, T cell follicular helper, regulatory T cells (Tregs), and T cell gamma delta); naïve and memory B cells; plasma cells; resting and activated NK cells; monocytes; macrophages M0, M1, and M2; resting and activated dendritic cells; resting and activated mast cells; eosinophils; and neutrophils. The CIBERSORT *p* value and root mean squared error (RMSE) were calculated for each tissues. Only samples with a CIBERSORT *p* value < 0.05 were enrolled. Eventually, there were 144 normal samples from GTEx and 6 normal samples and 141 tumor samples from TCGA were eligible in the study (Supplementary Figure 1).

### 2.3. Connectivity Map (CMap) Analysis

CMap (https://portals.broadinstitute.org/cmap/, RRID:SCR_015674) was used to find connections between drugs and genes [18]. Up tag file and down tag file were uploaded into the quick query of the tool web. The value of connectivity score fluctuated between -1 and 1, and a high negative connectivity score manifested that the drug reversed the expression of the query signature.

### 2.4. Statistical Analyses

For each immune cell phenotypes, we calculated the quartile, median, and third quartile of the normal and tumor groups. The Wilcoxon signed-rank test was used to compare the different TIICs. Correlation analysis was performed by package “corrplot”of R. Correlations between clinicopathological characters and proportions of TIICs were realized by the Wilcoxon signed-rank test and visualized by GraphPad Prism 8.0.1 (RRID:SCR_002798). The Lasso regression model was built by package “glmnet” and “survival” of R. Univariate and multivariate cox analyses and forestplot were completed by “survival” and “forestplot” of R. GO analysis between the high- and low-risk groups was conducted by “clusterProfiler,” “http://org.Hs.eg.db/,” “enrichplot,” and “ggplot2.” Gene set enrichment analysis (GSEA) was performed to get the core genes of top sets (RRID:SCR_003199) [19, 20]. All tests were two-tailed *p* value, and *p* value < 0.05 was considered significant. Survival was evaluated with the hazard ratio (95% confidence intervals).

## 3. Results

### 3.1. Different Proportions of Immune Cell Infiltration in Normal and Tumor Tissues

**Figure 1:**
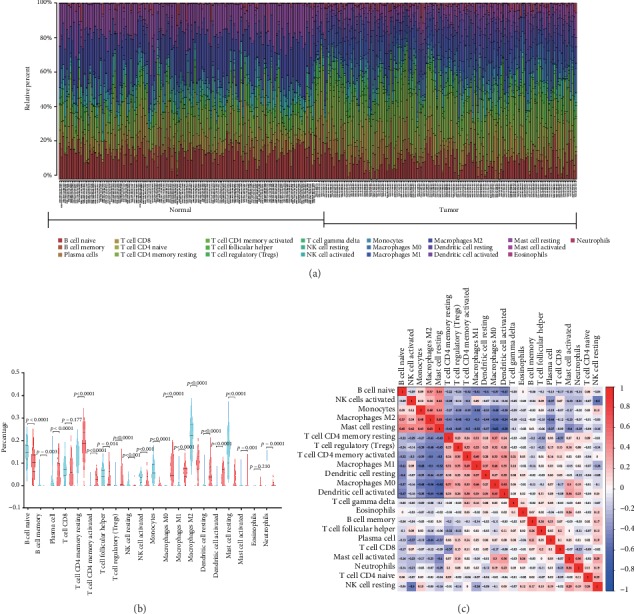
Relative distributions of infiltrated immune cells between normal and tumor tissues and correlation of TIICs in EC. (a) Distribution of 22 infiltrated immune cells in 150 normal tissues and 141 tumor tissues of EC. Each bar represented the relative proportion of infiltrated immune cells of one tissue. (b) Violin plot of infiltrated immune cells between normal and tumor tissues. The blue color represented normal tissue, and the red represented tumor tissues. Inner violin plot showed the quartile, median, and third quartile. (c) Correlation analysis among each TIIC in esophagus cancer. The blue color represented negative correlation, and the red represented positive correlation.

We evaluated the proportions of 22 TIICs in 150 normal tissues and 141 tumor tissues of EC by the CIBERSORT algorithm. From the results of Figures 1(a) and 1(b) and Table 1, 20 TIICs had a statistic difference between normal and tumor tissues. Setting∣log2FC∣ > 2, *p* < 0.05 as significant difference cutoff, the proportions of B cell memory, monocytes, and resting mast cells were significantly decreased in EC, while the proportions of activated T cell CD4 memory, regulatory T cells (Tregs), macrophage M0, macrophage M1, resting dendritic cells, and activated dendritic cells were significantly increased.

To illuminate the potential relationship of TIICs, we performed correlation analysis among them. From Figure 1(c), there were positive correlations among the percentage of naïve B cell, monocytes, macrophage M2, and resting mast cells. Similarly, there also existed positive correlations among the proportions of resting T cell CD4 memory, regulatory T cells (Tregs), activated T cell CD4 memory, macrophage M1, resting dendritic cells, macrophage M0, and activated dendritic cells. Also, there were negative correlations between the groups naïve B cell, monocytes, macrophage M2, and resting mast cells and the groups resting T cell CD4 memory, regulatory T cells (Tregs), activated T cell CD4 memory, macrophage M1, resting dendritic cells, macrophage M0, and activated dendritic cells.

### 3.2. Correlations between Clinicopathological Characters and Proportions of TIICs in EC

**Figure 2:**
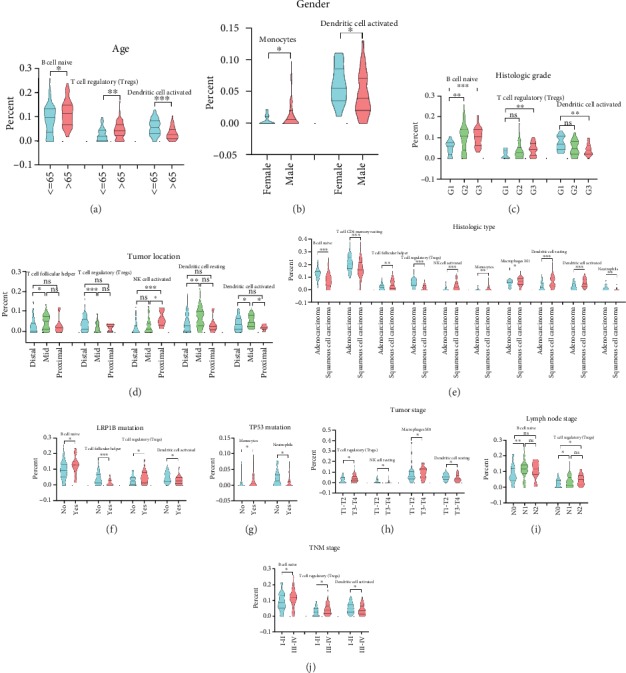
Correlations between clinicopathological features and proportions of TIICs in EC. Comparison of significantly different percentages of TIICs for different age (a), gender (b), histologic grade (c), tumor location (d), histologic type (e), LRP1B mutation status (f), TP53 mutation (g), tumor stage (h), lymph node stage (i), and TNM stage (j).

For basic information of EC patients, the proportions of naïve B cell and regulatory T cells (Tregs) were higher in older patients (age > 65), while the proportion of activated dendritic cells was significantly reduced in older male patients (Figures 2(a) and 2(b)). The percentage of monocytes was slightly increased in male patients (Figure 2(b)). The higher the histologic grade was, the higher proportions of naïve B cell and regulatory T cells (Tregs) were found in EC (Figure 2(c)), while the trend was opposite in activated dendritic cells (Figure 2(c)). When it came to the tumor location, the percentages of T cell follicular helper, resting dendritic cells, and activated dendritic cells rose in mid portion (Figure 2(d)). Besides, the proportion of regulatory T cells (Tregs) was decreased in mid portion and activated NK cells were upregulated in proximal portion (Figure 2(d)). With regard to the histologic type, naïve B cell, resting T cell CD4 memory, regulatory T cells (Tregs), and neutrophils had higher percentages in adenocarcinoma of the esophagus, while T cell follicular helper, activated NK cells, monocytes, macrophage M1, resting dendritic cells, and activated dendritic cells rose in proportion in squamous cell carcinoma of the esophagus (ESCC) (Figure 2(e)). As for mutation in EC, the percentages of naïve B cell and regulatory T cells (Tregs) were higher, and T cell follicular helper and activated dendritic cells were lower for LRP1B mutation (Figure 2(f)). The proportion of monocytes increased, and neutrophils were cut for TP53 mutation (Figure 2(g)). There were more proportions of Tregs and macrophage M0, while less of resting NK cells and resting dendritic cells in higher tumor stages (Figure 2(h)). Furthermore, the proportions of naïve B cell and Tregs increased in the higher lymph node stage and TNM stage (Figures 2(i) and 2(j)).

### 3.3. Prognostic Value of Proportions of TIICs in EC

**Figure 3:**
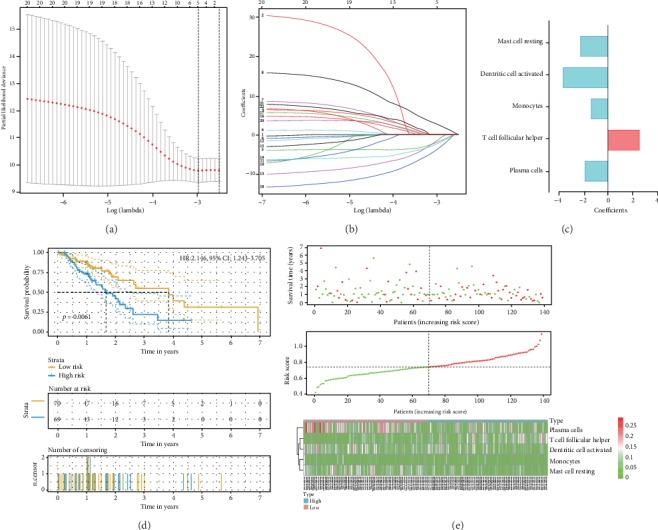
Establishment of the risk score model with TIICs. (a, b) The process of constructing the lasso regression model with size and coefficients by multivariate cox regression. (c) Coefficients of TIICs in the risk model. (d) The survivorship curve of risk model stratified by TIICs. (e) Distributions of five immune cell infiltration involved in the risk model, survival status, and survival time in the low- and high-risk groups.

**Figure 4:**
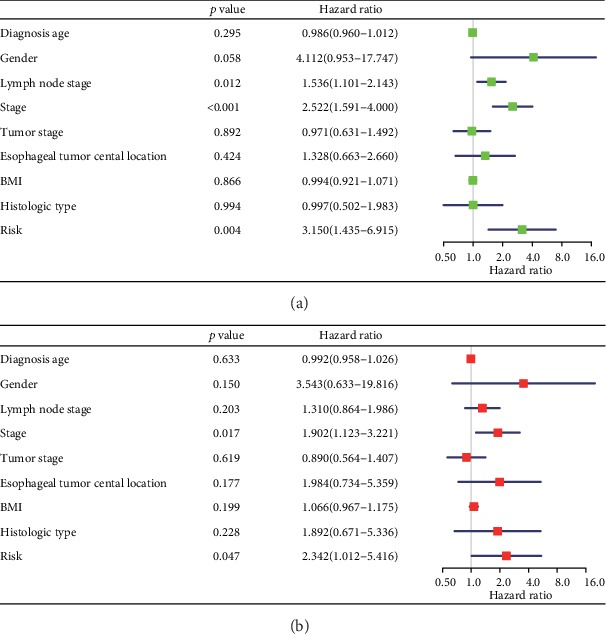
Univariate (a) and multivariate (b) cox regression analyses of clinicopathological features and the risk model.

To investigate the prognostic value of TIICs, we performed univariate cox regression analysis. The result of Supplementary Figure 2 showed that one proportion of TIICs cannot predict the outcome. Thus, we constructed a lasso regression model to improve prognostic capacity. Figures 3(a) and 3(b) showed the process of building the risk model. A five-cell immune infiltration-related risk model was built: −1.86∗plasma + 2.56∗T cell follicular helper − 1.37∗monocytes − 3.64∗activated dendritic cells − 2.24∗resting mast cells (immune cells in the risk model mean the proportions of immune cell infiltration in EC) (Figure 3(c)). Then, we divided patients into two groups: the high-risk group (risk score > median value of all patients) and the low-risk group (risk score ≤ median value). The survivorship curve (Figure 3(d)) indicated that patients in the high-risk group had significantly worse overall survival than that in low-risk group (HR: 2.146, 95% CI: 1.243-3.705, *p* = 0.0061).  Figure 3(e) showed that the distributions of five immune cell infiltration involved in risk model, survival status and survival time in low- and high-risk group, indicating the great diagnostic performance of the risk model. As a validation of GSE19417 in an indirect manner, the risk score of poorly differentiated group was higher than that of the well-differentiated group (*p* = 0.025). Besides, the risk score of patients with more than 5 positive lymph nodes was higher than that in patients with only 1 positive lymph node (*p* = 0.033) (Supplementary Figure 3). It is reported that the increasing number of positive nodes is related to poor outcome of EC [21]. Furthermore, from the results of univariate (HR: 3.150, 95% CI: 1.435-6.915, *p* = 0.004, Figure 4(a)) and multivariate (HR: 2.342, 95% CI: 1.012-5.416, *p* = 0.047, Figure 4(b)) cox analyses, the risk score was the independent risk factor for EC.

### 3.4. Identification of Two Candidate Compounds for the Treatment of EC Based on CMap Analysis

**Figure 5:**
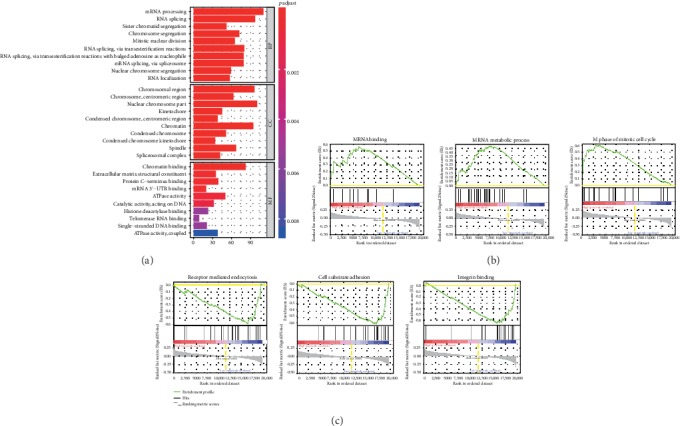
Gene ontology and gene set enrichment analyses between low- and high-risk groups stratified by the risk model. (a) The top ten gene ontology-enriched annotations between the low- and high-risk groups. (b) The top three enriched gene sets of the high-risk group. (c) The top three enriched gene sets of the low-risk group. BP: biological process; CC: cellular component; MF: molecular function.

**Figure 6:**
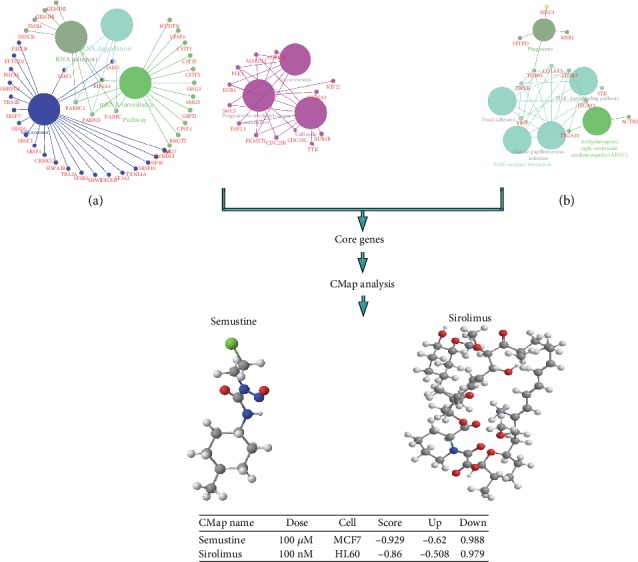
Identification of two candidate compounds for the treatment of EC based on CMap analysis. (a) Identification of core genes from the top three enriched gene sets of the high-risk group. (b) Identification of core genes from the top three enriched gene sets of the low-risk group.

GO analysis was conducted to illuminate the differential enrichment signals between the low- and high-risk groups stratified by immune cell infiltration of EC. The top ten enriched signals were shown in the matter of biological process (BP), cellular component (CC), and molecular function (MF). And the terms “mRNA processing,” “chromosomal region,” and “chromatin binding” were the most enriched signals of BP, CC, and MF, respectively (Figure 5(a)). Then, GSEA was performed to identify the core genes.  Figure 5(b) showed the most enriched sets mRNA binding, mRNA metabolic process, and M phase of mitotic cell cycle in the high-risk group, while Figure 5(c) displayed the most enriched gene sets receptor-mediated endocytosis, cell substrate adhesion, and integrin binding in the low-risk group. The core genes of high-risk group obtained from mRNA binding, mRNA metabolic process, and M phase of the mitotic cell cycle were visualized in Figure 6(a), and the core genes of low-risk group from gene sets, receptor mediated endocytosis, cell substrate adhesion, and integrin binding, were revealed in Figure 6(b). Then, 11 upregulation core genes (LSM3, LSM5, PABPC1, PABPC3, PABPC4, EIF4A3, SMC3, ESPL1, CDC25B, CCNA2, and AURKA) and 1 downregulation core gene (THBS3) were uploaded into CMap as upregulated tags and downregulated tags, respectively. Two compounds (Semustine and Sirolimus) might be the candidate compounds for the treatment of EC.

## 4. Discussion

To date, malignant tumors remain the main killer of human health [1]. The focus of the research in the past with regard to the tumor development is often on the tumor cells themselves. In 1989, Paget first proposed the theory of “seed and soil,” which has been widely recognized and extended since its introduction [22]. The theory holds that the occurrence and development of tumors not only depend on the change in tumor cytogenetics and epigenetics but also link to the tumor microenvironment as a “fertile soil” for the growth and breeding of malignant seeds [22, 23]. In normal tissues, the microenvironment is an important barrier for defense against tumors. However, the inhibitory function in tumor cells would be reduced by recruiting integrated fibroblasts, regulating antitumor immune cells, secreting immunosuppressive molecules, and thus transforming into tumor microenvironment suitable for tumor growth [24]. It is just the interaction and coevolution of tumor cells and their microenvironment that promote the production of tumors. In recent years, increasing attention has been given to the tumor microenvironment in terms of the potential response to therapy.

In EC, it is also reported that tumor microenvironment has a close relationship with tumor growth. The cells, including NK cells, T cells, B cells, plasma cells, monocytes, macrophages, dendritic cells, mast cells, eosinophils, and neutrophils, have all been reported to have exhibited significant association with EC treatment [7, 10, 25–32]. The elevation or reduction of these different types of TIICs in tumor microenvironment is commonly associated with poor prognosis of EC. Nevertheless, prior studies only focused on the density and clinical value of one or two TIICs in EC, lacking systematic analysis of all TIICs with a different density. In this study, following the operation of CIBERSORT algorithm, we found that 20 immune-infiltrating cells had statistic difference simultaneously between normal and tumor tissues. Of note, the proportions of B cell memory, monocytes, and resting mast cells were significantly decreased in EC, while the proportions of activated T cell CD4 memory, Tregs, macrophage M0, macrophage M1, resting dendritic cells, and activated dendritic cells were significantly increased.

Tregs are a subpopulation of CD4+ T lymphocytes. It could inhibit the antitumor immunity and assist in tumor immune escape through secreting immunosuppressive factors, directly killing or inhibiting effector cell proliferation, as well as affecting T cell activation [33, 34]. The clinical research also points that the high-density infiltration of Tregs often indicates the poor clinical outcomes. In severely immunodeficient mice, the repeated reinfusion of patient-derived CD3+CD25 T cells could prevent tumor growth. For the negative regulation of Tregs against tumor immune function, in line with this, it was found that the reinfusion of Tregs could reverse such protection [35, 36]. Similarly, Nishikawa et al. found that leukocytes isolated from patients with melanoma and ovarian cancer had responded to selective tumor antigens only after removal of Tregs [37]. In this study, by analyzing the correlations between clinicopathological characters and TIICs, we found that Treg density infiltration in EC patients had a close association with age, histologic grade, tumor location, histologic type, LRP1B mutation, tumor stage, lymph node stage, and TNM stage. In fact, it has been acknowledged that Tregs increased according to the EC progression [38]. In ESCC, IL32 expression and Treg infiltration were found to play an important synergistic role in tumor growth and invasion; the combination of which was one of poor independent factors [39]. In operative specimens, generally, the frequency of local Tregs negatively correlated with the pathological response and overall patient survival [40]. However, up to now, there are still no relevant reports on Tregs and LRP1B, and it remains to be studied whether the poor prognosis of patients in the high age group can be considered from the perspective of Treg infiltrating density.

Additionally, from the results in Figure 2, it was shown that macrophages M0 and M1 differed greatly in different tumor stages and histologic types, respectively. Unlike Tregs, infiltrated macrophages are developed from a precursor of bone marrow monocytes that are activated into different subtypes by stimulation signals in different microenvironments [41, 42]. Studies have shown that macrophages induced immune disability through promoting angiogenesis, inducing tumor metastasis, provoking chemotherapy tolerance, and interacting with the other immune cells or raising other immune inhibitory cells [43]. Macrophage is one of the important factors in tumor microenvironments that cause the poor prognosis of patients. But for the moment, it is relatively frequent on the research about the relationship between macrophages and ESCC. Compared with adenocarcinoma, there is no basic data to support whether the infiltration of macrophages in ESCC is relatively high. In addition to macrophages, dendritic cells are also associated with a variety of clinicopathological features in EC. Dendritic cell is one of the most potent antigen-presenting cells [44]. It was realized that the impaired immune function and the decreased number of dendritic cells were the significant causes for the pathogenesis and progression of EC [45]. In EC, a research once has pointed that LRP1B possessed the recurrent copy-number variant character, significantly promoting cancer cell proliferation, migration, and invasion [46]. A deep research of the relationship between dendritic cells and LRP1B gene is a great necessity since there are no such reports until now.

Considering the low survival rate of EC patients, subsequently, univariate cox regression analysis was performed in exploring the prognostic value of these immune cell infiltration. It was found that one proportion of immune cell infiltration cannot predict the outcome. According to the lasso regression model, it was shown that the joint detection of plasma, T cell follicular helper, monocytes, activated dendritic cells, and resting mast cell infiltration exhibited great performance for its diagnostic. In EC, as indicated above, for instance, Svensson et al. guessed that the antitumoral effects on time to recurrence (TTR) and OS were largely dependent on a functional interplay between T and B lymphocytes or plasma cells [47]. In the study by Lu et al., the higher number of CD1a dendritic cells had a correlation with significantly improved OS of patients with ESCC [48]. Nonetheless, due to the more complexity of TIICs, the full consideration of the link between TIICs and tumor prognosis is still in the blank stage. In this study, we have not only addressed the issue but also found that the risk score was an independent risk factor for EC. The risk factor presented a great link with EC prognosis including age, sex, location of tumor, histologic type, tumor stage, and nodal invasion. Overall, in assessing the EC prognosis, it is necessary to fully evaluate the above factors. In this regard, while multiple reports in the literature have linked the presence of different cases with its prognosis [49], many others have found no exact assessment for considering all involved factors. Thus, in such circumstances, the acquisition of an independent risk factor would favor tumor prognosis assessment. In such later cases, sometimes, for simple and convenient assessment, the risk score could be chosen as an independent risk factor.

Additionally, the study found that mRNA binding, the mRNA metabolic process, and the M phase of mitotic cell cycle were enriched in the high-risk group, while receptor-mediated endocytosis, cell substrate adhesion, and integrin binding were enriched in the low-risk group. Through the assessment of these differential enrichment signals, 11 upregulation core genes (LSM3, LSM5, PABPC1, PABPC3, PABPC4, EIF4A3, SMC3, ESPL1, CDC25B, CCNA2, and AURKA) and 1 downregulation core gene (THBS3) were identified. LSM3 was found downregulated in cervical cancer, correlating with progression free survival [50, 51]. In colorectal cancer, LSM3 also was significantly associated with lymphatic metastasis [52], but in EC, there is still no report. Similarly, there is just a report in breast cancer that the high expression of PABPC1 would promote cancer tumorigenesis and resistance [53] and in lung adenocarcinoma, it might be involved in tumor development [54]. In follicular thyroid cancer, it was identified as a mutated cancer driver gene. Except for PABPC1, PABPC3 was also found as a cancer driver gene in follicular thyroid cancer [55]. For PABPC4, it exhibited significant differences in expression in immune cell-infiltrating breast tumors, hopefully regarding as a candidate for its diagnostics and therapy [56]. Besides, Liu et al. pointed that PABPC4 likely played a role in the pathogenesis of colorectal cancer [57]. In various types of cancers, numerous studies have now documented a link between EIF4A3, SMC3, ESPL1, CDC25B, CCNA2, or AURKA and their response to therapy [58–63]. For the downregulation core gene THBS3, conversely, it was expressed at significantly high levels in osteosarcoma, which was a predictor of worse OS at diagnosis [64]. All in all, there is few report of the above genes in EC.

In order to find candidate drugs that may provide novel insights into tumorigenesis therapeutic advancements for EC based on immune cell infiltration, we performed CMap analysis. As a result, we found two compounds Semustine and Sirolimus that were promising candidates for the treatment of EC. Semustine is a nitrosourea antitumor drug, which belongs to nonspecific cell cycle drug. Summarizing the indications, Semustine has a good curative effect on malignant melanoma, lymphoma, brain tumor, and lung cancer. However, up to now, there are few clinical studies of Semustine in patients with EC. The finding thus is expected to expand the application range of Semustine. In addition to such chemotherapeutic, the use of immunosuppressants is an important change in the way the cancer is treated. For EC, at present, most immunosuppressants are still in the clinical research stage. For instance, pembrolizumab is currently in Phase Ib study with PD-L1-positive esophageal cancer patients, exhibiting tolerable toxicity and effective antitumor effect [65]. In clinical II phase, nivolumab monotherapy has achieved good results in patients with advanced EC of which the objective response rate is 17.2% and the disease control rate is 42% [66]. Although in this study, for Sirolimus, we have identified it as a potential candidate inhibitor for EC; a large number of trials are needed to evaluate its antitumor activity and safety.

Finally, we have to admit some limitations of the study. Owing to the insufficiency of clinical data and independent validation cohort, we cannot further prove our finding. We have endeavoured to collect as many samples as possible from public database. Regretfully, we just found a validation to preliminarily verify the value of the risk score. Besides, we set about collecting sample of EC in our own institute. Our findings would be further validated with basic biology experiment.

## 5. Conclusion

In EC, 20 TIICs were identified as having a statistical difference between normal and tumor tissues. These TIICs had great links with clinicopathological characters. A five-TIIC risk score model was built to guide prognosis of EC. Based on the enrichment analysis of stratification by the risk score, two candidate compounds Semustine and Sirolimus were found as therapeutic strategy for EC. In general, the proportions of TIICs are likely to be important to the progression, prognosis, and treatment of EC.

## Figures and Tables

**Table 1:** Different proportions of immune cell infiltration in normal and tumor tissues of esophagus cancer.

Elements	Normal mean	Tumor mean	|Log_2_FC|	*p* value
Naïve B cell	0.150328828	0.099252886	-0.59894074	2.69*E*-15
Memory B cell	0.004501406	0.000624709	-2.849119332	0.003169
Plasma cell	0.045761521	0.065299383	0.512934359	3.22*E*-05
T cell CD8	0.076262723	0.072047936	-0.082020934	0.177424
Naïve T cell CD4	0.000169422	0	NA	0.092744
Resting T cell CD4 memory	0.129593083	0.188880046	0.54348158	7.24*E*-15
Activated T cell CD4 memory	0.00149565	0.032217892	4.429017635	1.74*E*-36
T cell follicular helper	0.04635218	0.038316743	-0.274662245	0.014469
Regulatory T cell (Treg)	0.005591945	0.038123896	2.769273551	1.38*E*-28
T cell gamma delta	0	0.00076106	NA	0.038445
Resting NK cell	0.019864511	0.011368435	-0.805159562	0.000221
Activated NK cell	0.034988158	0.027034051	-0.372088999	0.000235
Monocytes	0.065933699	0.013084405	-2.333167736	5.27*E*-34
Macrophage M0	0.00158835	0.08482624	5.738909999	3.37*E*-50
Macrophage M1	0.011738194	0.059927909	2.35201761	3.74*E*-36
Macrophage M2	0.220429513	0.102609075	-1.10315907	2.46*E*-33
Resting dendritic cell	0.008666269	0.048631755	2.48841577	1.73*E*-24
Activated dendritic cell	0.006626912	0.048122149	2.860292337	3.26*E*-34
Resting mast cell	0.154389795	0.038572188	-2.00094452	8.25*E*-37
Activated mast cell	0.004146638	0.01388375	1.74338341	0.001248
Eosinophil	0.002143682	0.00235641	0.136499567	0.210273
Neutrophil	0.009427524	0.014059083	0.576551742	1.02*E*-06

## Data Availability

The data used to support the findings of this study are included within the article.

## Supplementary Materials

Supplementary Materials Supplementary Figure 1 shows the flow diagram of process. Supplementary Figure 2 displays univariate Cox regression analysis of 22 infiltrated immune cells in EC. Supplementary Figure 3 shows the validation of the risk score in an indirect manner.

## References

[1] Bray F., Ferlay J., Soerjomataram I., Siegel R. L., Torre L. A., Jemal A. (2018). Global cancer statistics 2018: GLOBOCAN estimates of incidence and mortality worldwide for 36 cancers in 185 countries. *CA: a Cancer Journal for Clinicians*.

[2] Domper Arnal M. J., Ferrández Arenas Á., Lanas Arbeloa Á. (2015). Esophageal cancer: risk factors, screening and endoscopic treatment in Western and eastern countries. *World Journal of Gastroenterology*.

[3] Miller K. D., Siegel R. L., Lin C. C. (2016). Cancer treatment and survivorship statistics, 2016. *CA: a Cancer Journal for Clinicians*.

[4] Domingues P., González-Tablas M., Otero Á. (2016). Tumor infiltrating immune cells in gliomas and meningiomas. *Brain, Behavior, and Immunity*.

[5] Garnelo M., Tan A., Her Z. (2017). Interaction between tumour-infiltrating B cells and T cells controls the progression of hepatocellular carcinoma. *Gut*.

[6] Bense R. D., Sotiriou C., Piccart-Gebhart M. J. (2016). Relevance of tumor-infiltrating immune cell composition and functionality for disease outcome in breast cancer. *Journal of the National Cancer Institute*.

[7] Xu B., Chen L., Li J. (2016). Prognostic value of tumor infiltrating NK cells and macrophages in stage II+III esophageal cancer patients. *Oncotarget*.

[8] Huang H., Zhang G., Li G., Ma H., Zhang X. (2015). Circulating CD14^+^HLA-DR^−/low^ myeloid-derived suppressor cell is an indicator of poor prognosis in patients with ESCC. *Tumour Biology*.

[9] Tsutsumi S., Saeki H., Nakashima Y. (2017). Programmed death-ligand 1 expression at tumor invasive front is associated with epithelial-mesenchymal transition and poor prognosis in esophageal squamous cell carcinoma. *Cancer Science*.

[10] Izawa S., Mimura K., Watanabe M. (2013). Increased prevalence of tumor-infiltrating regulatory T cells is closely related to their lower sensitivity to H2O2-induced apoptosis in gastric and esophageal cancer. *Cancer Immunology, Immunotherapy*.

[11] Baxevanis C. N., Papamichail M., Perez S. A. (2013). Immune classification of colorectal cancer patients: impressive but how complete?. *Expert Opinion on Biological Therapy*.

[12] Grizzi F., Basso G., Borroni E. M. (2018). Evolving notions on immune response in colorectal cancer and their implications for biomarker development. *Inflammation Research*.

[13] Newman A. M., Liu C. L., Green M. R. (2015). Robust enumeration of cell subsets from tissue expression profiles. *Nature Methods*.

[14] Goldman M., Craft B., Hastie M., Brooks A., Zhu J., Haussler D. The UCSC Xena platform for public and private cancer genomics data visualization and interpretation. *BioRxiv*.

[15] Lonsdale J., Thomas J., Salvatore M. (2013). The genotype-tissue expression (GTEx) project. *Nature Genetics*.

[16] Ritchie M. E., Phipson B., Wu D. (2015). *Limma* powers differential expression analyses for RNA-sequencing and microarray studies. *Nucleic Acids Research*.

[17] Peters C. J., Rees J. R. E., Hardwick R. H. (2010). A 4-gene signature predicts survival of patients with resected adenocarcinoma of the esophagus, junction, and gastric cardia. *Gastroenterology*.

[18] Subramanian A., Narayan R., Corsello S. M. (2017). A next generation connectivity map: L1000 platform and the first 1,000,000 profiles. *Cell*.

[19] Subramanian A., Tamayo P., Mootha V. K. (2005). Gene set enrichment analysis: a knowledge-based approach for interpreting genome-wide expression profiles. *Proceedings of the National Academy of Sciences*.

[20] Mootha V. K., Lindgren C. M., Eriksson K. F. (2003). PGC-1alpha-responsive genes involved in oxidative phosphorylation are coordinately downregulated in human diabetes. *Nature Genetics*.

[21] Rice T. W., Ishwaran H., Hofstetter W. L. (2017). Esophageal cancer: associations with (pN+) lymph node metastases. *Annals of Surgery*.

[22] Paget S. (1989). The distribution of secondary growths in cancer of the breast. 1889. *Cancer Metastasis Reviews*.

[23] Langley R. R., Fidler I. J. (2011). The seed and soil hypothesis revisited-the role of tumor-stroma interactions in metastasis to different organs. *International Journal of Cancer*.

[24] Junttila M. R., de Sauvage F. J. (2013). Influence of tumour microenvironment heterogeneity on therapeutic response. *Nature*.

[25] Li Y., An J., Huang S., He J., Zhang J. (2015). Esophageal cancer-derived microvesicles induce regulatory B cells. *Cell Biochemistry and Function*.

[26] Wouters M. C. A., Nelson B. H. (2018). Prognostic significance of tumor-infiltrating B cells and plasma cells in human cancer. *Clinical Cancer Research*.

[27] Zhu Y., Li M., Bo C. (2017). Prognostic significance of the lymphocyte-to-monocyte ratio and the tumor-infiltrating lymphocyte to tumor-associated macrophage ratio in patients with stage T3N0M0 esophageal squamous cell carcinoma. *Cancer Immunology, Immunotherapy*.

[28] Yang W., Yu J. (2008). Immunologic function of dendritic cells in esophageal cancer. *Digestive Diseases and Sciences*.

[29] Fakhrjou A., Niroumand-Oscoei S. M., Somi M. H., Ghojazadeh M., Naghashi S., Samankan S. (2014). Prognostic value of tumor-infiltrating mast cells in outcome of patients with esophagus squamous cell carcinoma. *Journal of Gastrointestinal Cancer*.

[30] Zhang Y., Ren H., Wang L. (2014). Clinical impact of tumor-infiltrating inflammatory cells in primary small cell esophageal carcinoma. *International Journal of Molecular Sciences*.

[31] Liu S., Huang L. P., Lin Z. (2019). Association between the time of neutrophils to the lowest and prognosis of patients with esophageal squamous cell carcinoma treated with non-operative therapy. *Zhonghua Zhong Liu Za Zhi*.

[32] Xie X., Luo K. J., Hu Y., Wang J. Y., Chen J. (2016). Prognostic value of preoperative platelet-lymphocyte and neutrophil-lymphocyte ratio in patients undergoing surgery for esophageal squamous cell cancer. *Diseases of the Esophagus*.

[33] Nishikawa H., Sakaguchi S. (2014). Regulatory T cells in cancer immunotherapy. *Rinshō Ketsueki*.

[34] Zitvogel L., Tanchot C., Granier C., Tartour E. (2013). Following up tumor-specific regulatory T cells in cancer patients. *Oncoimmunology*.

[35] Bates G. J., Fox S. B., Han C. (2006). Quantification of regulatory T cells enables the identification of high-risk breast cancer patients and those at risk of late relapse. *Journal of Clinical Oncology*.

[36] Curiel T. J., Coukos G., Zou L. (2004). Specific recruitment of regulatory T cells in ovarian carcinoma fosters immune privilege and predicts reduced survival. *Nature Medicine*.

[37] Nishikawa H., Jäger E., Ritter G., Old L. J., Gnjatic S. (2005). CD4+ CD25+ regulatory T cells control the induction of antigen-specific CD4+ helper T cell responses in cancer patients. *Blood*.

[38] Sander F. E., Nilsson M., Rydström A. (2017). Role of regulatory T cells in acute myeloid leukemia patients undergoing relapse-preventive immunotherapy. *Cancer Immunology, Immunotherapy*.

[39] Nabeki B., Ishigami S., Uchikado Y. (2015). Interleukin-32 expression and Treg infiltration in esophageal squamous cell carcinoma. *Anticancer Research*.

[40] Vacchelli E., Semeraro M., Adam J., Dartigues P., Zitvogel L., Kroemer G. (2015). Immunosurveillance in esophageal carcinoma: the decisive impact of regulatory T cells. *Oncoimmunology*.

[41] Davies L. C., Jenkins S. J., Allen J. E., Taylor P. R. (2013). Tissue-resident macrophages. *Nature Immunology*.

[42] Qian B. Z., Pollard J. W. (2010). Macrophage diversity enhances tumor progression and metastasis. *Cell*.

[43] Adeegbe D. O., Nishikawa H. (2013). Natural and induced T regulatory cells in cancer. *Frontiers in Immunology*.

[44] Tian H., Li W. (2017). Dendritic cell-derived exosomes for cancer immunotherapy: hope and challenges. *Annals of Translational Medicine*.

[45] Chen S. R., Luo Y. P., Zhang J. K. (2004). Study on immune function of dendritic cells in patients with esophageal carcinoma. *World Journal of Gastroenterology*.

[46] Chang J., Tan W., Ling Z. (2017). Genomic analysis of oesophageal squamous-cell carcinoma identifies alcohol drinking-related mutation signature and genomic alterations. *Nature Communications*.

[47] Svensson M. C., Warfvinge C. F., Fristedt R. (2017). The integrative clinical impact of tumor-infiltrating T lymphocytes and NK cells in relation to B lymphocyte and plasma cell density in esophageal and gastric adenocarcinoma. *Oncotarget*.

[48] Lu L., Pan K., Zheng H. X. (2013). IL-17A promotes immune cell recruitment in human esophageal cancers and the infiltrating dendritic cells represent a positive prognostic marker for patient survival. *Journal of Immunotherapy*.

[49] Vendrely V., Launay V., Najah H., Smith D., Collet D., Gronnier C. (2018). Prognostic factors in esophageal cancer treated with curative intent. *Digestive and Liver Disease*.

[50] Lyng H., Brøvig R. S., Svendsrud D. H. (2006). Gene expressions and copy numbers associated with metastatic phenotypes of uterine cervical cancer. *BMC Genomics*.

[51] Tan M. S., Chang S. W., Cheah P. L., Yap H. J. (2018). Integrative machine learning analysis of multiple gene expression profiles in cervical cancer. *PeerJ*.

[52] Xie N., Yao Y., Wan L., Zhu T., Liu L., Yuan J. (2017). Next-generation sequencing reveals lymph node metastasis associated genetic markers in colorectal cancer. *Experimental and Therapeutic Medicine*.

[53] Dong H., Wang W., Mo S. (2018). Long non-coding RNA SNHG14 induces trastuzumab resistance of breast cancer via regulating PABPC1 expression through H3K27 acetylation. *Journal of Cellular and Molecular Medicine*.

[54] Murugesan S. N., Yadav B. S., Maurya P. K., Chaudhary A., Singh S., Mani A. (2018). Expression and network analysis of YBX1 interactors for identification of new drug targets in lung adenocarcinoma. *Journal of Genomics*.

[55] Erinjeri N. J., Nicolson N. G., Deyholos C., Korah R., Carling T. (2018). Whole-exome sequencing identifies two discrete druggable signaling pathways in follicular thyroid cancer. *Journal of the American College of Surgeons*.

[56] Kostianets O., Antoniuk S., Filonenko V., Kiyamova R. (2012). Immunohistochemical analysis of medullary breast carcinoma autoantigens in different histological types of breast carcinomas. *Diagnostic Pathology*.

[57] Liu D., Yin B., Wang Q. (2012). Cytoplasmic poly(a) binding protein 4 is highly expressed in human colorectal cancer and correlates with better prognosis. *Journal of Genetics and Genomics*.

[58] Zhang S., Leng T., Zhang Q., Zhao Q., Nie X., Yang L. (2018). Sanguinarine inhibits epithelial ovarian cancer development via regulating long non-coding RNA CASC2-EIF4A3 axis and/or inhibiting NF-*κ*B signaling or PI3K/AKT/mTOR pathway. *Biomedicine & Pharmacotherapy*.

[59] Kraft B., Lombard J., Kirsch M. (2019). SMC3 protein levels impact on karyotype and outcome in acute myeloid leukemia. *Leukemia*.

[60] Wang D., Zhu H., Guo M. (2018). Expression and prognostic value of cell-cycle-associated genes in gastric adenocarcinoma. *BMC Gastroenterology*.

[61] Al-Matouq J., Holmes T. R., Hansen L. A. (2019). CDC25B and CDC25C overexpression in nonmelanoma skin cancer suppresses cell death. *Molecular Carcinogenesis*.

[62] Deng J. L., Xu Y. H., Wang G. (2019). Identification of potential crucial genes and key pathways in breast cancer using Bioinformatic Analysis. *Frontiers in Genetics*.

[63] Yen C.-C., Chen S. C., Hung G. Y. (2019). Expression profile-driven discovery of AURKA as a treatment target for liposarcoma. *International Journal of Oncology*.

[64] Dalla-Torre C. A., Yoshimoto M., Lee C. H. (2006). Effects of THBS3, SPARC and SPP1 expression on biological behavior and survival in patients with osteosarcoma. *BMC Cancer*.

[65] Doi T., Piha-Paul S. A., Jalal S. I. (2018). Safety and antitumor activity of the anti-programmed death-1 antibody pembrolizumab in patients with advanced esophageal carcinoma. *Journal of Clinical Oncology*.

[66] Kudo T., Hamamoto Y., Kato K. (2017). Nivolumab treatment for oesophageal squamous-cell carcinoma: an open-label, multicentre, phase 2 trial. *The Lancet Oncology*.

