# Supplementary material for: Comprehensive Analysis of Tumor-Infiltrating Immune Cells and Relevant Therapeutic Strategy in Esophageal Cancer
Source: Dis Markers. 2020 May 11;2020:8974793. doi: 10.1155/2020/8974793 (PMC7238334; doi:10.1155/2020/8974793)
Supplement: Supplementary Materials — Supplementary Figure 1 shows the flow diagram of process. Supplementary Figure 2 displays univariate Cox regression analysis of 22 infiltrated immune cells in EC. Supplementary Figure 3 shows the validation of the risk score in an indirect manner. [file 8974793.f1.pdf]

Supplementary Figure 1

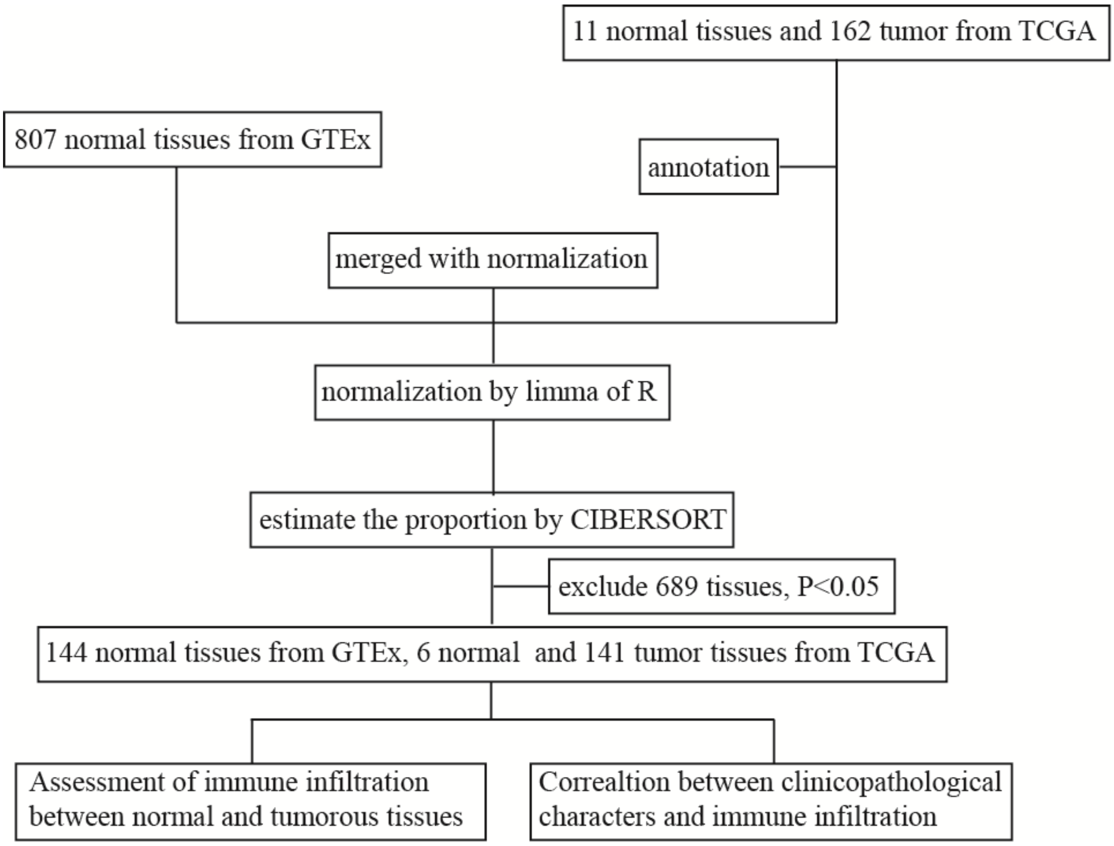

Supplementary Figure 1. the flow diagram of process.

Supplementary Figure 2

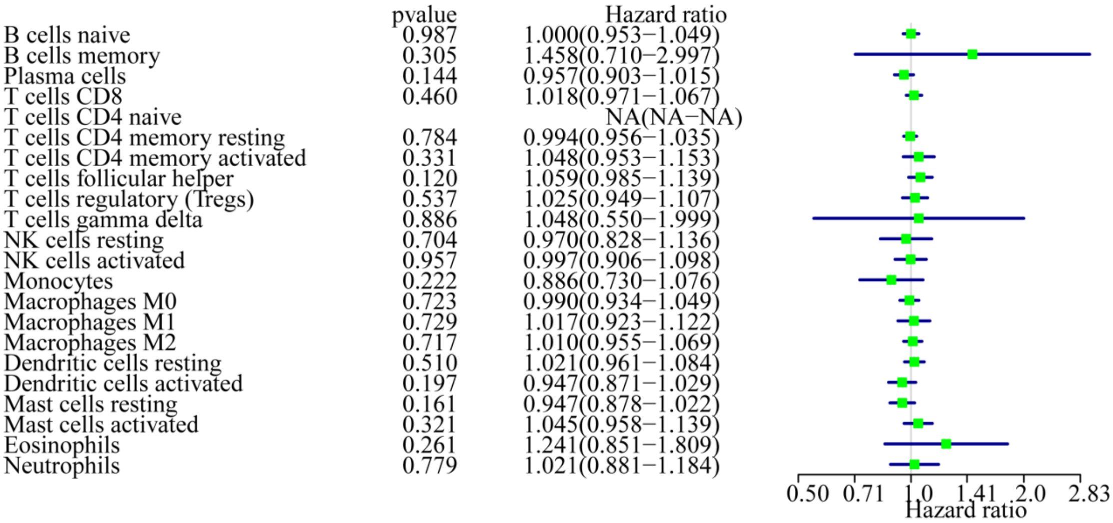

Supplementary Figure 2. Univariate Cox regression analysis of 22

infiltrated immune cells in EC. Hazard ratio was shown with 95% confidence intervals.

Supplementary Figure 3

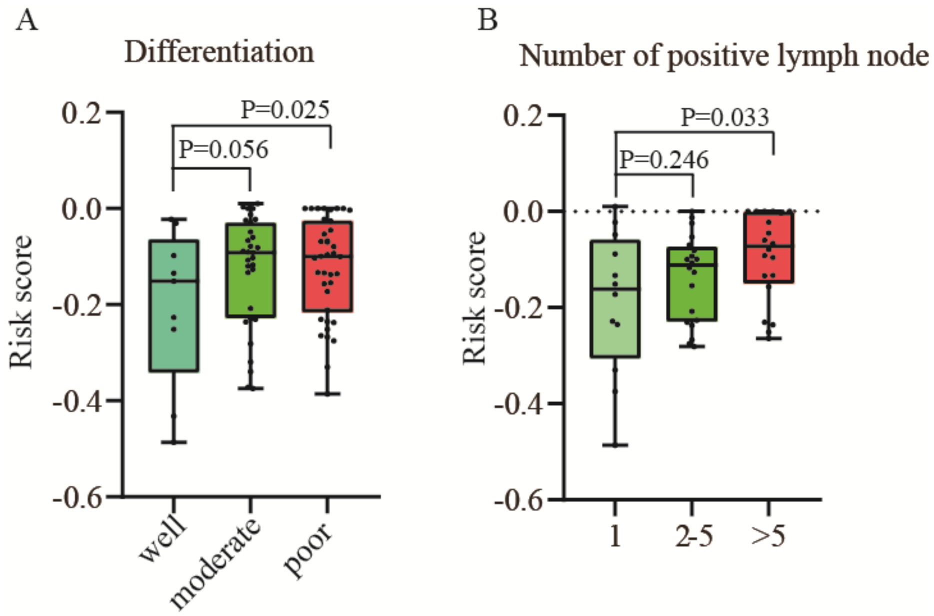

Supplementary Figure 3. (A) The distribution of the risk score in different differentiation degrees in the validation cohort. (B) The distribution of the risk score with various number of positive lymph nodes in the validation cohort.
